# Comparison of Analgesic and Anti-Inflammatory Effects of Kale Extract Versus Ibuprofen After Impacted Mandibular Third Molar Surgery: A Randomized, Double-Blind Split-Mouth Clinical Trial

**DOI:** 10.3390/nu16223821

**Published:** 2024-11-07

**Authors:** Vuttinun Chatupos, Sansanee Neelawatanasook, Tidanut Sangutai, Atit Khanutwong, Pattaranee Srichairatanakool, Wachiraporn Tipsuwan, Onsaya Kerdto, Narisara Paradee, Pimpisid Koonyosying, Somdet Srichairatanakool

**Affiliations:** 1 Department of Oral and Maxillofacial Surgery, Faculty of Dentistry, Chiang Mai University, Chiang Mai 50200, Thailand; vuttinun.ch@cmu.ac.th (V.C.); ching_6561@hotmail.com (S.N.); bsayonara9@gmail.com (T.S.); atitttt.k@gmail.com (A.K.); 2 Department of Anesthesiology, School of Medicine, University of Phayao, Phayao 56000, Thailand; pattaranee.sr@up.ac.th; 3 Division of Biochemistry, School of Medical Sciences, University of Phayao, Phayao 56000, Thailand; wachiraporn.t@up.ac.th; 4 Department of Biochemistry, Faculty of Medicine, Chiang Mai University, Chiang Mai 50200, Thailand; onsaya35@gmail.com (O.K.); narisara.p@cmu.ac.th (N.P.); pimpisid.k@cmu.ac.th (P.K.)

**Keywords:** third molars, visual analogue scale, kale, Brassica oleracea α-amylase, matrix metalloproteinase 9, transforming growth factor beta2

## Abstract

Background/Objective: We assessed the analgesic and anti-inflammatory effects of kale extract (500 mg anthocyanin equivalent) in patients after mandibular molar surgery. Methods: In our randomized clinical trial, postoperative subjects (n = 20) aged 18–25 years old took kale extract or ibuprofen (400 mg) capsules for 7 days, or vice versa, after surgical removal of each impacted tooth. Their pain intensity was then assessed using a visual analogue scale (VAS). Moreover, salivary α-amylase (AA) activity, matrix metalloproteinase 9 (MMP-9) and transforming growth factor beta2 (TGF-β_2_) concentrations were measured. Levels of VAS and AA decreased 7 days after the first and second molar extractions in the two treatment groups. Results: The kale extract was more effective than ibuprofen. MMP-9 and TGF-β_2_ levels were reduced on days 4 and 7 following the two extractions in the kale group, whereas they were reduced on days 4 and 7 following the first extraction in the ibuprofen group. There was a positive correlation between MMP-9 and TGF-β_2_. Thus, the consumption of the kale extract exerted analgesic and anti-inflammatory effects during the postoperative period in patients who had undergone molar extractions. In conclusion, anthocyanin-abundant kale extract is preferable when administered in a postoperative course and could reduce the need for a prescription of ibuprofen.

## 1. Introduction

Impacted teeth are those that cannot erupt due to a physical barrier and are often considered for removal. If they remain within the jaw, their follicles can potentially become cystic. Accordingly, the removal of impacted teeth can be needed to prevent periodontal disease, dental caries, pericoronitis, root resorption, odontogenic cysts and tumors, and jaw fractures requiring orthodontic treatment. Their removal can also optimize periodontal healing. Pain is the most prevalent complication experienced by patients following dentoalveolar surgery and must be controlled; otherwise, it will likely result in patient dissatisfaction, discomfort and systemic sequels (e.g., tachycardia, hypertension, improper nutrition and central sensitization) [1,2]. Ibuprofen (400 mg) is the most common non-steroidal anti-inflammatory drug (NSAID) used for the management of acute dentoalveolar pain and other pains associated with wisdom and mandibular molar removal, but it can also cause nausea, vomiting, headaches, dizziness and decreased platelet aggregation [3,4]. In addition to any potential adverse effects, ibuprofen can inhibit bone repair at the early stages of endochondral ossification by suppressing cyclooxygenases (COXs) to inhibit prostaglandin (PGs) (e.g., PGE_2_) synthesis. Thereby, it can inhibit α-amylase to reduce neurogenic pain stimuli, enhance matrix metalloproteinase (MMPs) mRNA expression, promote production and activity by enhancing healing effects, attenuate endothelial type IV procollagen synthesis and prevent fibroblasts’ formation.

The visual analog scale (VAS) is a tool used to measure the intensity or frequency of pain from none (0) to extreme amounts (10), wherein subjects will mark their pain intensity on the VAS of 0–10 [5]. In addition, sensitive biomarkers in saliva are evaluated in response to symptoms and diseases, including lactoferrin for oral mucositis [6], IL-6 and IL-1α for active oral chronic graft-versus-host disease [7], IL-1β, MMP-8, TNF-α and PGE_2_ for periodontal disease [8,9], chromogranin A and α-amylase for pain or displeasure during and after orthodontic treatment [10], α-enolase, IL-18 and kallikrein-13 for burning mouth syndrome [11], colony-stimulating factor-1 for periodontitis and caries [12] and α-amylase for post-surgical removal of mandibular molars. Subsequently, α-amylase, MMP-9 and TGF-β_2_ are of significant interest for investigations involving saliva samples.

On a molar basis, human saliva contains certain bioactive proteins, such as α-amylase (AA), MMPs and transforming growth factors (TGFs), which, when functioning properly in the oral cavity, can be used for the diagnosis of dental diseases. For instance, salivary AA is a simple effective biomarker for the evaluation of pain intensity in patients during orthodontic tooth movement, as well as postoperatively in the removal of mandibular third molars [5,13]. MMP-9 is a type of gelatinase that regulates cell differentiation during osteogenesis, bone healing and angiogenesis. Besides periodontal parameters, MMP-9 can be manifested as a local inflammatory response and investigated in saliva and gingival tissues [14,15]. It has been found to have increased in the tissue samples obtained from apical periodontitis patients undergoing third molar extractions [16] and in the saliva of oral pathology subjects [17,18]. In addition, levels of MMP-9 mRNA expression and production were found to have significantly increased on days 3 and 7 in Zoledronic acid-treated rats [19]. Moreover, MMP3 and MMP9 mRNA expressions were increased in the tissues surrounding replanted teeth after extraction [20]. Transforming growth factor-beta (TGF-β) is synthesized by osteoblasts, osteocytes and chondrocytes. It then links bone resorption and formation during remodeling by enhancing osteoblast proliferation, suppressing osteoblast differentiation, stimulating bone formation and inhibiting bone resorption, all of which have been proposed to be anti-apoptotic effects in osteoblasts [21,22]. Importantly, TGF-β_2_ stimulates extracellular matrix (ECM) synthesis, as well as deposition and dentin apposition, during tooth development [23,24]. For example, TGF-β_2_ gene expression was decreased when the healing of tooth extraction sockets was delayed [25]. Furthermore, TGF-β1, β2 and β3 gene expressions were increased in saliva from patients with oral submucous fibrosis, and the increased levels were restored following the consumption of a cocktail (antioxidants, nutrients and micro-nutrients) or through the intravenous injection of hyaluronidase [26]. While serum AA, MMP9 and TGF-β_2_ concentrations are well-established biomarkers for diagnosing and monitoring symptoms and diseases, their salivary concentrations are emerging as sensitive indicators of pain, inflammation and gingival healing, respectively, in individuals who have undergone molar extraction(s). Hormesis is a biphasic adaptive dose-response of cells that are exposed to stimuli (e.g., mild stress, free radicals and toxic substances) and is induced to protect the biological system against subsequently large and potentially lethal stresses [27]. Plants contain hormetic nutrients such as polyphenols, flavonoids and vitamins that can protect and relieve oxidative diseases or syndromes by activating cells to resist severe stress through signaling pathways or causing toxicity, depending on the concentrations used [28,29]. For instance, low-dose polyphenols in synergy with probiotics show biphasic dose-response antioxidant and anti-inflammatory activities by targeting the nuclear factor erythroid 2-related factor 2 (Nrf2) pathway and vitagenes, which improve gut bioavailability, prevent gastrointestinal diseases and inhibit the onset/progression of neurological disorders [30,31].

Nowadays, there are increasing levels of interest in the discovery of plant-derived extract remedies with analgesic, anti-inflammatory or wound healing properties, such as anthocyanins, which have been investigated less in this regard. Anthocyanins (ACNs) are found ubiquitously in cabbage, broccoli, kale, black currants and grapes. They potently exert antioxidant and anti-inflammatory effects [32,33]. For instance, the consumption of an ACN-rich plant extract could exert antioxidant, analgesic and anti-inflammatory properties in the muscle tissues [34] of hyperlipidemic apolipoprotein E-deficient mice [35,36] and rats with temporomandibular joint inflammation [36]. Importantly, ACNs exhibit anti-aging properties in human dermal fibroblast cells that have been exposed to ultraviolet irradiation [37].

Interestingly, kale (*Brassica oleracea* L., Brassicaceae Family) is known as the queen of greens and a superfood that is rich in vitamin C, total carotenoids, fiber, minerals and antioxidative compounds [38,39]. The predominant phytochemicals present in red curly kale leaves include flavonols (e.g., quercetin, kaempferol and kaempferol-3-sinapoyl-diglucoside-7-diglucoside), anthocyanins (e.g., cyanidin-3-sinapoyl-feruloyl-diglucoside-5-glucoside), phenolic acids (e.g., sinapic acid, ferulic acid and disinapoyl-diglucoside) [40] and glucosinolates (e.g., sinigrin) [41]; however, these contents are often decreased after the kale has undergone blanching, frozen storage and boil-in-bag heat treatments [41]. In particular, kaempferol, and some kaempferol glycosides, exert anti-inflammatory, anti-osteoporotic, analgesic and antiallergic activities [42], whereas sinigrin has demonstrated antioxidant, anti-inflammatory and wound healing activities [43]. Ethanolic extracts of the leaves of the *Brassica villosa* subspecies drepanensis have been reported to inhibit the activities of α-amylase, α-glucosidase and lipase, and to express anti-inflammatory activities in lipopolysaccharide-stimulated RAW 264.7 cells [44]. Moreover, certain dishes in Bangladeshi cuisine, such as “Panch phoron”, that are made from *Brassica nigra* and are comprised of catechin hydrate, para-coumaric acid, vanillic acid and syringic acid, have been reported to exhibit anti-inflammatory activity and postoperative analgesia in male mice [45]. Furthermore, the oral administration of *Allium sativum*, *Brassica oleracea* and *Aloe barbadensis* extracts restored gastric and duodenum ulcerations in indomethacin-induced Wistar rats, indicating certain mid-gut anti-inflammatory and healing properties [46]. At present, the anti-pain and anti-inflammatory effects of *B. oleracea*-derived products on patients after mandibular molar extraction or surgery have not yet been fully studied. The outcomes of this study validate our attempts to promote ACN-rich kale as a functional food with analgesic and anti-inflammatory activities when compared with the reference ibuprofen in postoperative patients based on their VAS scores and levels of salivary biomarkers. The aim of this study is to compare the efficacy of kale extract consumption on pain intensity and salivary α-amylase, as well as MMP-9 and TGF-β_2_ levels, in people subjected to mandibular lower third molar extractions. This was done in an effort to decrease the requirement of prescribing ibuprofen to patients postoperatively for the purpose of pain management.

## 2. Materials and Methods

### 2.1. Chemicals, Reagents and Equipment

Authentic standards, including cyanidin-3-glucoside (C3G), keracyanin-3-rutinoside (K3R), callistaphin glucoside (CPG), 3,4,5-trihydroxybenzoic acid or gallic acid (GA), peonidin-3-glucoside (P3G), malvidin-3-galactoside (M3G), quercetin (Q) and quercetin-3-glucoside (Q3G), 2,2′-azino-bis(3-ethylbenzothiazoline-6-sulfonic acid (ABTS), Folin-Ciocalteu reagent, and 6-hydroxy-2,5,7,8-tetramethylchroman-2-carboxylic acid (or Trolox), were obtained from Sigma-Aldrich Chemicals Company Ltd., Saint Louis, MO, USA. A Megazyme reagent kit for the analysis of α-amylase (AA) activity was obtained from the NEOGEN Corporation, Wicklow, Ireland. Sandwich-type enzyme-linked immunosorbent assay (ELISA) kits for MMP-9 and TGF-β_2_ were supplied by ABBEXA Company Ltd., Bar Hill, Cambridge, UK. All chemicals and were of HPLC or AnalaR grade.

### 2.2. Drugs

Articaine hydrochloride/epinephrine solution (Septacaine^®^ and epinephrine, 1:100,000 dilution, Septodont Inc. Company, Lancaster, PA, USA), ibuprofen (400 mg tablet, Heidi-400) and paracetamol (500 mg tablet, Tylenol-500) were purchased from a drug store in the Faculty of Dentistry, Chiang Mai University, Thailand. Ibuprofen tablets were crushed with a clean porcelain tablet mortar and pestle, transferred to similarly colored gelatin capsules (400 mg drug content) as the kale extract and stored in virtually labeled bottles that had been coded with a small red dot at the bottom.

### 2.3. Preparation and Analysis of Encapsulated Kale Extract

#### 2.3.1. Preparation of Water Extract of Kale Leaves

Fresh curly kale (*Brassica oleracea* var. sabellica L.) was harvested from fields located in Tambon Mae Hae, Amphur Mae Wang, Chiang Mai Province, Thailand in November 2022. A big crop of kale (200 kg fresh weight) was dried in a hot-air oven at 50–55 °C for 24 h. All the dried leaves (16.9 kg) were then ground using a powder grinder machine at the same factory. Afterward, the kale powder was placed and extracted in hot water (70 °C) (1 kg in 10 L) to allow the powder to dissolve for 10 min and 10% (w/w) maltodextrin (good grade) was added. It was then filtered through a clean fabric sheet and centrifuged twice at 6000 rpm for 15 min each time [47].

#### 2.3.2. Production of Kale Extract Capsules

A total of 50 L of kale extract was cooled in a vacuum freeze dryer machine until one part of the liquid formed pure crystalline ice and the remainder of the sample was frozen-concentrated into a glassy state. The formed ice was then removed by sublimation under a vacuum (10^−4^ atmosphere) and at a low temperature (−30 °C) until the product was dried. Finally, kale granules were encapsulated and kept in white plastic bottles with caps.

#### 2.3.3. Analysis of Chemical Compositions

##### Total Anthocyanins Content (TAC)

Herein, the TAC was determined using the pH differential method [48]. Kale extracts (200 µL) were dissolved in deionized water (DI) and incubated with 25 mM, pH 1.0 potassium chloride (800 µL) or 0.4 M, pH 4.5 sodium acetate (800 µL) for 30 min. The absorbance (A) value was then measured spectrophotometrically at 520 and 700 nm against the reagent blank. Accordingly, the TAC was calculated and reported as µg TAC/g of dry weight of the kale extract using Equation (1) as follows:

ACN pigment (C3G equivalents, mg/L) = (A × MW × DF × 10^3^)/ε × 1 (1)

Note: A = (A_520nm_ − A_700nm_) value at pH 1.0 − (A_520nm_ − A_700nm_) value at pH 4.5; molecular weight (MW) of C3G = 449.2 g/moL; DF = dilution factor; molar extinction coefficient (ε) for C3G = 26,900 L/mol*cm; l = pathlength in cm; and 10^3^ = a conversion from g to mg.

##### Anthocyanin Derivatives

Anthocyanin compounds were identified and quantified by using a high-performance liquid chromatography/diode array detection/electrospray ionization–mass spectrometry (HPLC/DAD/ESI-MS) method [49,50]. The instrument system (Agilent Technologies 1100 Series, Deutschland GmbH, Waldbronn, Germany) consisted of a quaternary pump, an online vacuum degasser, an autosampler, a thermo-regulated column compartment, a photodiode array (PDA) detector and mass spectrometer (MS) detector. In terms of the analysis, kale extract granules were constituted in 1.0 mL of a mixture comprised of solvent A (acetonitrile) and solvent B (10 mM formate buffer pH 4.0) (1:1, *v*/*v*), then injected (20 μL), fractionated on a column (LiChroCART RP-18e, 150 mm × 4.6 mm, 5 µm particle size; Purospher STAR, Merck, Darmstadt, Germany), thermally regulated at 40 °C, eluted with the mobile phase solvents A and B at a flow rate of 1.0 mL/minute under the gradient program of 100% solvent B (0% solvent A) for an initial period of 5 min, 0–20% solvent A from 5 to 10 min, 20% solvent A from 10 to 20 min, 20–40% solvent A from 20 to 60 min and 40% solvent A for 3 min, and the A detected with the PDA set at 270 and 520 nm. The single quadrupole MS analysis was done in the positive ESI mode at 70 eV energy and the spectra were acquired with a mass to charge ratio (*m*/*z)* ranging from 100 to 700, while the temperatures of the ion source and the interface were set at 150 °C and 230 °C, respectively. The capillary temperature was set to 320 °C, the pressure of the nebulizing, drying and collision nitrogen gas was set to 60 psi and the drying gas flow rate was set to 13 L/minute. Capillary voltages were set to 3500 V (positive) and 150 V (negative). The oven temperature was programmed as follows: 80 °C (held for 3 min), ramped to 110 °C at 10 °C/minute (held for 5 min), increased to 190 °C (held for 3 min), ramped to 220 °C at 10 °C/minute (held for 4 min) and increased to 280 °C at 15 °C/minute (held for 13 min). Accurate mass measurements were performed by employing the auto mass calibration method using an external mass calibration solution (ESI-L Low Concentration Tuning Mix; Agilent calibration solution B). Herein, the limit of detection (LOD), limit of quantitation (LOQ) and recovery value were found to be 0.5 mg/kg, 1.20 mg/kg and 70–110%, respectively. The chromatographic and mass spectrometric analysis and prediction of the chemical formula, including the exact mass calculation, were performed by Mass Hunter software version B.04.00 built to 4.0.479.0 (Agilent Technology, Deutschland GmbH, Waldbronn, Germany). In addition, available standards including C3G, K3R, CPG, P3G, M3G and Q3G were run to position the eluted compounds, and their concentrations present in the kale extract were calculated.

##### Total Phenolic Content (TPC)

The TPC in the kale extract granules was measured using the Folin–Ciocalteu method [51]. The kale extract (100 µL) was mixed with 10% (*v*/*v*) Folin-Ciocalteu reagent (200 µL) diluted in DI and incubated at 25 °C for 4 min. Accordingly, 700 mM sodium carbonate (800 µL) was added to the mixture and incubated at 25 °C for 30 min. The solution (200 µL) was transferred to 96-well plates and measured spectrophotometrically at 765 nm. GA was dissolved in DI to prepare a standard curve. The TPC was reported as mg equivalents of gallic acid standard per g of dry extract (mg GAE/g extract).

##### Total Flavonoid Content (TFC)

The kale extract (250 µL) was mixed with 10% aluminum chloride (50 µL), 1 M potassium acetate (50 µL) and DI (2.15 mL). It was then incubated in the dark at 25 °C for 30 min [52]. The solution (200 µL) was transferred to 96-well plates and measured spectrophotometrically at 415 nm. Various concentrations of Q were prepared in absolute methanol to prepare a standard curve. The TFC was reported as mg equivalents of quercetin standard per g of dry extract (mg QE/g extract).

#### 2.3.4. Determination of Free-Radical Scavenging Activity

The Trolox equivalent antioxidant capacity (TEAC) of the kale extract was estimated by the ABTS radical cation (ABTS^+^) decolorization assay [53]. The working ABTS^+^ solution consisted of 7 mM stock ABTS^+^ solution and 2.45 mM potassium persulfate solution (1:1), which was prepared and stored at 25 °C for 12 h before being used. The working ABTS^+^ solution was diluted with DI to an absorbance of 0.70 ± 0.02 at 734 nm. The kale extract (10 µL) was mixed with the diluted ABTS^+^ solution (1.0 mL) and incubated in the dark at 25 °C for exactly 6 min. The A was measured at 764 nm. The results were reported as mg equivalents of Trolox standard per g of dry weight of extract (mg TE/g extract). Trolox was dissolved in absolute ethanol at different concentrations and used to construct a standard curve.

### 2.4. Ethics

Ethical approval for this project was granted by the Human Experimentation Committee of the Faculty of Dentistry, Chiang Mai University, Chiang Mai, Thailand (Certificate Number: 65/2022, Date: 16 December 2022). All patients were fully informed about the particulars of the study and willingly provided their signatures on consent forms before they participated in the study. The investigations were carried out following the rules of the Declaration of Helsinki of 1975 (https://www.wma.net/what-we-do/medical-ethics/declaration-of-helsinki/) (accessed on 4 January 2023), revised in 2013, and meet national and international guidelines.

### 2.5. Clinical Trial Registry

The clinical study protocol was registered, reviewed and approved by the International Standard Randomized Controlled Trial Number (ISRCTN) Committee of the British Medical Council (reference number: ISRCTN48573608, Date: 17 March 2024), as can be viewed via the following link (https://www.isrctn.com/ISRCTN48573608) (accessed on 4 January 2023). This study was conducted in accordance with the reporting guidelines of the Consolidated Standards of Reporting Trials (CONSORT) 2010 [54].

### 2.6. Study Design and Participants

#### 2.6.1. Subject Selection

Volunteers were patients who had undergone lower third molar tooth extraction procedures at the Oral and Maxillofacial Surgery Clinic (OPD 3), Faculty of Dentistry, Chiang Mai University, Chiang Mai, Thailand. The patients were fully informed that their personal information (such as first name, family name, gender, hospital number and home address) would not be reported or/and published anywhere and that they were allowed to leave the study whenever they chose. A randomized controlled trial was then carried out. Data were collected from the clinical records of the OPD 3 for indication of extraction and orthopantomograms for the presence, site, angulation, depth and ramus relationship of impaction of the third molar. An impacted tooth is one that fails to fully erupt into the dental arch within the usual range of expected time. The most common impacted teeth are the maxillary and mandibular third molars, followed by the maxillary canines and mandibular premolars.

##### Indications and Contraindications

Indications for lower third molar extractions include recurrent pericoronitis, unrestorable caries or caries extending into the pulp, horizontal or mesioangular lower molars causing disto-cervical caries in the lower second molars, and third molars with odontogenic cysts or tumors. Contraindications of molar extractions are at high risk for ankylosis, complex root morphology, hypercementosis and inferior alveolar nerve damage, and they are not recommended for patients undergoing intravenous bisphosphonate treatment or radiotherapy [55].

##### Inclusion Criteria

Subjects were selected as patients within an age range of 18–25 years who were of healthy status (American Society of Anesthesiologists: ASA class I and II) [56] and those presenting bilaterally impacted lower third molars with mesioangular impaction requiring surgical extraction. In addition, they were free from pathological conditions and denied having any history of chronic illnesses and/or drug allergies. Additionally, all subjects had just undergone a molar extraction.

##### Exclusion Criteria

(1) Patients who were pregnant, heavy smokers or allergic to ibuprofen, mepivacaine or epinephrine-related drugs. (2) Patients who had presented any pre-existing medical conditions such as hypertension, diabetes, liver disease, kidney disease, blood clotting disorders and gastritis. (3) Patients who potentially had pericoronitis or pain related to impacted lower third molars or facial/jaw regions on the day of surgery. (4) Patients who had taken medication or dietary supplements that could impact the wound healing process. (5) Patients who experienced psychological or communication disorders.

##### Potential Discontinuation Criteria

(1) Patients could give up or withdraw from the study at any time. (2) Patients who experienced difficulties or complexities pertaining to their tooth extractions within 30 min of the procedure. (3) Patients who experienced severe post-extraction pain and who were unable to adequately manage that pain with the provided pain-relief medication alone or those who required additional pain-relief medication beyond the course of the prescription. (4) Patients who experienced postoperative complications such as uncontrolled bleeding, infections, paresthesia or alveolar osteitis (dry socket).

#### 2.6.2. Sample Size Calculation

The sample size (n) was calculated using the method previously described by Seymour and coworkers [57], using the STATA version 16.0 software (StataCorp, LLC, College Station, TX, USA) and Equation (2), shown below:

n = 2σ^2^(Z_α/2_ + Z_β_)^2^ ÷ (μ_1_ − μ_2_)^2^ (2)

where Z is a constant, the mean difference (μ_1_ − μ_2_) of virtual analog scale (VAS) = 3.0, standard deviation (SD, σ) = 3.6, confidence level = 95%, power of test = 80, alpha (α) = 0.05 and beta (β) = 0.20. According to the above calculation, the sample size required for the present study was 10 subjects per group. Subsequently, thirty-two subjects were further divided into two groups: the kale extract and ibuprofen groups (n = 16 each). Nevertheless, we decided to increase the sample size to a total of 20 individuals by including additional participants. This study was then divided into two parts in which the clinical part involved an assessment of pain levels using VAS, and the laboratory part involved the collection of saliva specimens for the measurement of AA activity as well as MMP-9 and TGF-β_2_ concentrations.

### 2.7. Randomization

Using the balanced block randomization method, a person who was not involved in this study (neither a dentist/researcher nor a patient) was kindly asked to randomly divide all the 20 subjects into two groups who would receive two different treatments after each molar extraction. This resulted in equal sample sizes across groups over time and prevented severe imbalances in sample allocation with respect to both known and unknown confounders. The random allocation within the blocks was done by Excel software version 2410 (Microsoft Office Professional Plus 2019, Microsoft Corporation, Redmond, WA, USA) shared license by Chiang Mai University, Thailand. Accordingly, the randomization of 20 subjects (A–T) proceeded by allocating random permutations of two treatments (1 = kale extract and 2 = ibuprofen) within each block size (randomly between 4 and 8).

### 2.8. Blinding and Allocation Concealment

The patients and the researcher who assessed the pain scores using VAS and analyzed the data were blinded to the group allocation and the use of kale extract or ibuprofen. The pain scale checklist was completed by an examiner who was blinded to the use of kale extract or ibuprofen. A highly experienced biochemist was also blinded to analyze the coded saliva samples. Moreover, the statistician who received the data of the two groups was not given any information about the type of intervention employed in each group.

### 2.9. Surgical Extraction of Third Molar Teeth

The medical histories of patients with impacted mandibular third molars were recorded and a physical examination was conducted prior to each tooth extraction procedure. The step-by-step procedure for tooth extraction, along with the potential risks associated with the treatment, was explained to the subjects. Each participant underwent extraction of one lower third molar on each side. One side was the experimental group, while the other side was the control group, with a one-month interval between sides. Local anesthesia was given to the subjects by intramuscular injection of a 4% articaine hydrochloride/epinephrine solution. Following that, flushing was achieved with an inferior alveolar nerve block (1.7 mL) and buccal infiltration (0.5 mL). The local anesthetic was administered and 10 min were allowed to pass for the action to take place. The tooth was then extracted using forceps and the wound was sutured with silk size 3-0. After the surgery, patients were instructed to bite down on a gauze pad for one hour and advised about postoperative care/discomfort. All surgeries were done by the same surgeon who administered either a simple extraction or surgical removal without bone removal or tooth section. The duration of the course of treatment was recorded after initiating the extraction or after the first incision was made. Each treatment then lasted until the placement of the last suture but did not exceed twenty minutes.

### 2.10. Study Intervention

Group 1 patients were instructed to take oral ibuprofen capsules (single dose 400 mg, four times daily after meals) after the first tooth extraction for 4 days and oral kale extract capsule (single dose 500 mg ACN equivalent, four times daily after meals) after the second tooth extraction for 4 days. Group 2 patients were instructed to take the oral single-dose kale extract after the first tooth extraction for 4 days and the oral single-dose ibuprofen after the second tooth extraction for 4 days. The participants were instructed to assess their pain levels using the VAS scoring tool and their saliva was collected for the analysis of AA activity and MMP-9 and TGF-β_2_ concentrations.

### 2.11. Postoperative Medication

Paracetamol tablets (500 mg size, Tylenol-500) were prescribed for the participants to take every 4 h when necessary for supplementary pain relief for 4 days. If they had a VAS score ≥ 5, they were asked to take 400 mg of ibuprofen.

### 2.12. Outcome Variables and Clinical Management

The predictor variable was a type of analgesic and anti-inflammatory agent used in the study and in the control group (kale extract or ibuprofen capsule, respectively). The primary outcomes of this study were the levels of self-reported pain intensity (based on VAS) and saliva biomarkers reported on days 1, 4 and 7 in the patients following the extraction of each impacted molar one month apart. It should be noted that the VAS evaluation and all tooth extractions were performed by a well-trained postgraduate student of adult dentistry. Other study variables included demographic information (including age and gender), operation time (defined as time between the first incision till flap closure) and the number of analgesics used during the first postoperative week.

### 2.13. Assessment of VAS

Practically, the VAS scoring tool is accessible on the Google document online site (https://1drv.ms/x/c/af36631b37a36afd/Ef1qozcbYzYggK_gCwAAAAABgKv7YzWwSyRq3KM_C5hFow) (accessed on 4 January 2023) and was used to evaluate the pain levels of the participants. Technically, the subjective VAS method employs a hypothetical 10 cm long line that depicts the subject’s pain level on a scale of 0–10, wherein 0 represents no pain and 10 represents the most conceivable suffering and pain. In the assessment, patients were asked to record their pain levels at 8:00 a.m. every day for one week, then mark it along these lines and measure the distance in millimeters between level 0 and the marked level. Notably, when the subjects used analgesic drugs, they were required to record the type, dosage and duration of time that the drugs were taken. To follow up, patients were appointed on days 4 and 7 after each surgery to evaluate the healing process and were told to come back if they faced persistent or increasing pain.

### 2.14. Laboratory Investigation

#### 2.14.1. Saliva Collection

Before the collection of saliva, patients were asked to abstain from eating and drinking for one hour. Saliva samples were collected from each patient at three different time points (before tooth extraction, on day 3 and on day 7 after tooth extraction) using a plastic micropipette tip, then transferred into a clean 15 mL polypropylene conical screw-cap tube and immediately delivered to the Iron Laboratory at the Department of Biochemistry, Faculty of Medicine, Chiang Mai University. The saliva specimens were filtered through a clean white gauze sheet, centrifuged at 1500 rpm at 4 °C, collected in 1.0 mL aliquots in microtubes (1.5 mL capacity) and then kept frozen in a freezer at −20 °C for up to 3 months for further analyses of salivary AA activity and MMP-9 and TGF-β_2_ concentrations, as will be described below.

#### 2.14.2. Determination of α-Amylase Activity

Salivary AA activity was determined using a Megazyme reagent kit (NEOGEN Corporation, Wicklow, Ireland) according to the manufacturer’s instructions, which is based on the Ceralpha procedure established by McCleary et al. [58]. In principle, an oligosaccharide substrate, defined as non-reducing-end blocked *p*-nitrophenyl maltoheptaoside (BPNPG7), was hydrolyzed by endo-acting α-amylase in the presence of excessive α-glucosidase. This was done to liberate glucose and free chromogenic *p*-nitrophenol (PNP), giving a yellow-colored product and a molar extinction (ε) value of ε_mM_ = 18.1 at λ_max_ of 400 nm. Before use, a vial of Amylase HR reagent containing 125 unit of α-glucosidase and 54.5 mg of BPNPG7 was reconstituted in 10.0 mL of distilled water. In the assay, 0.2 mL aliquots of Amylase HR reagent solution and saliva samples were pre-incubated separately at 40 °C for 5 min. Then, 0.2 mL of the pre-equilibrated saliva was added directly to the tubes containing Amylase HR reagent solution (0.2 mL) and the mixture was incubated at 40 °C for exactly 10 min (from the time of addition). At the end of the 10 min incubation period, 3.0 mL of stopping reagent (1% tri-sodium phosphate buffer, pH 11) was added exactly and the tube contents were stirred vigorously. Finally, the absorbance (A) values of the solutions and the reaction blank were read at 400 nm against distilled water. A standard curve was constructed by using different PNP concentrations. For quality control, the reagent kit indicated absolute specificity for α-amylase, a standard deviation value of 0.0189, standard errors of mean < 5% and a coefficient of variation of 4.05%. Thus, the α-amylase activity in Ceralpha units (CU/mL) was calculated by using the following equation:

α-Amylase activity (CU/mL) = (A_saliva_ − A_reagent_) × 4.7 × dilution of the saliva (3)

Accordingly, the international unit (IU) value was derived by multiplying the CU by 4.6.

#### 2.14.3. Quantifications of minMMP-9 and TGF-β_2_

Concentrations of salivary MMP-9 and TGF-β_2_ were determined using ELISA kits according to the manufacturer’s instructions. In terms of quality control, the human MMP-9 ELISA kits indicated a sensitivity of 0.1 ng/mL and coefficients of variation < 10% for intra-assay and inter-assay, while the human TGF-β_2_ ELISA kit indicated a sensitivity of <5.7 pg/mL and a coefficient of variation of <10% for intra-assay and <12% for inter-assay.

### 2.15. Statistical Analysis

**Figure 1:**
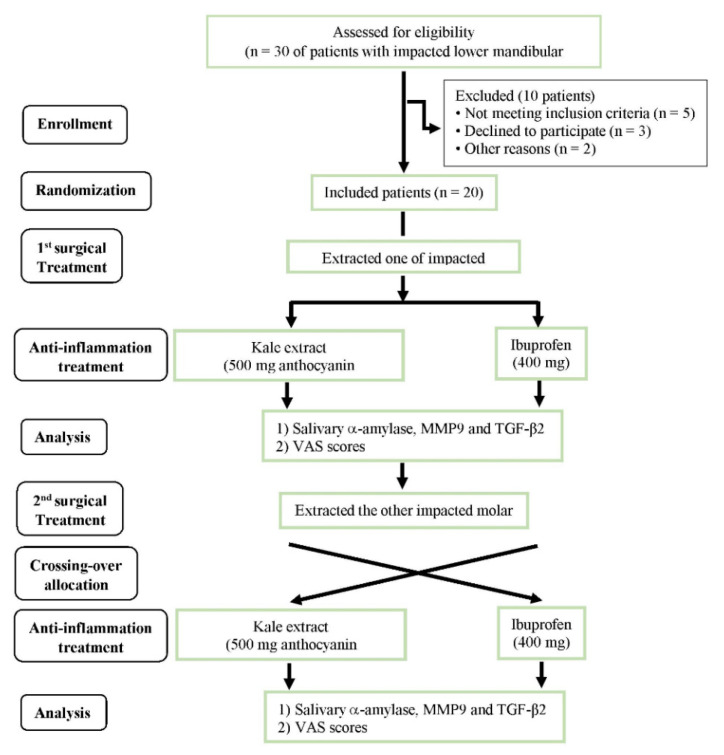
Flowchart of participants who were ineligible (participants excluded for not meeting inclusion criteria or those who were declined participation for unknown reasons) and eligible to take kale extract or ibuprofen. VAS and salivary levels of α-amylase, MMP-9 and TGF-β_2_ were then assessed. Abbreviations: MMP-9 = matrix metalloproteinase 9, TGF-β_2_ = transforming growth factor-beta 2, VAS = visual analogue scale.

Personal information and physiological/pathological records were collected at the time of the enrollment visit (day 0–1). The results of pain intensity were recorded at a mean of 1 cm on the VAS every day (11.00–11.45 a.m.) for 7 days and were analyzed using the Statistics Program for Social Sciences (SPSS) version 22 (IBM Inc., Armonk, NY, USA). They were then expressed as mean ± standard error of the mean (SEM). Similarly, AA activity and MMP-9 and TGF-β_2_ concentrations in the saliva samples that were collected on days 1, 4 and 7 post-molar extractions among the patients who had received kale extract and ibuprofen treatments were analyzed. Accordingly, a *p* < 0.05 value was considered significantly different. Parametric analysis of variance (ANOVA) and paired Student *t*-tests with post hoc or nonparametric Mann–Whitney U and Wilcoxson tests were used for comparisons of different times and groups. The relationship between VAS values, AA activity and MMP-9 and TGF-β_2_ concentrations were determined and compared using Spearman’s correlation coefficient at a 95% confidence level. This study complied with the appropriate guidelines/checklist of the STROBE protocol and the CONSORT flow diagram is shown in Figure 1.

## 3. Results

### 3.1. Kale Products

**Figure 2:**
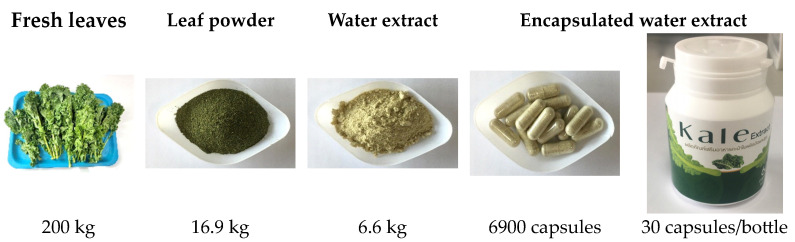
Depictions and yields of kale leaves, powder, water extract and capsules.

As is shown in Figure 2, a total of 200 kg of fresh kale leaves were processed sequentially to achieve a dry powder (16.9 kg), kale water extract (50 L liquid or 6.6 kg powder) and 6900 kale extract capsules (500 mg dry weight, 500 mg ACN each). Accordingly, the kale extract was analyzed in terms of its chemical composition and the kale extract capsules were orally administered to subjects, as described in the sections below.

### 3.2. Chemical Compositions

The findings issued from the analyses of the TPC, TFC and TAC are presented in Table 1. Using colorimetric assays, it was found that 1 g of kale extract granules contained a TPC of 24.92 ± 0.46 mg GAE, TFC of 3.09 ± 1.51 mg QE and TAC of 204.72 ± 1.71 µg CE (Table 1). In addition, the kale extract exerted ABTS^+^-scavenging activity in test tubes, possibly due to the actions of the phenolic, flavonoid and anthocyanin compounds.

**Figure 3:**
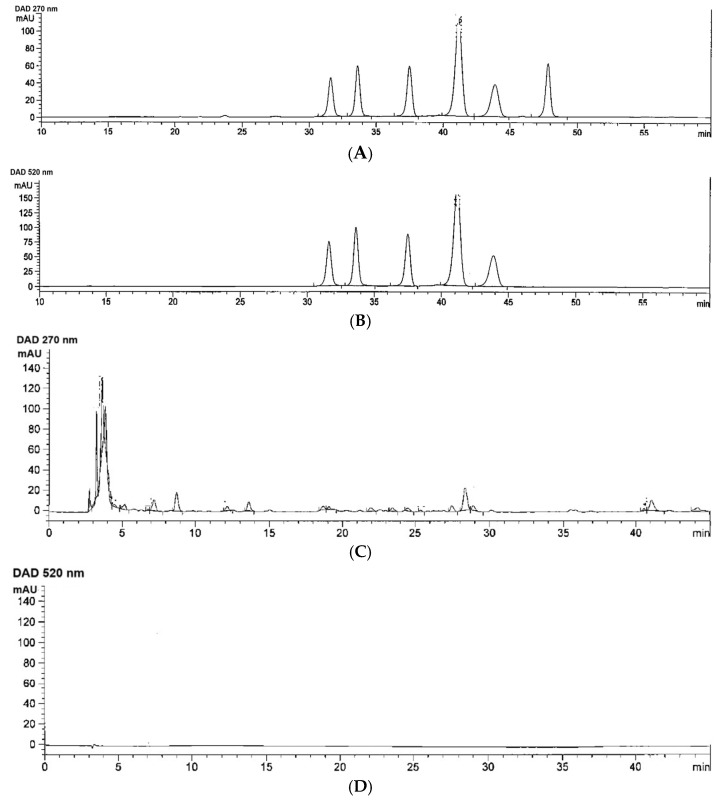
HPLC/DAD/ESI-MS analysis of authentic standards: cyanidin-3-glucoside, keracyanin-3-rutinoside, callistaphin glucoside, peonidin-3-glucoside, malvidin-3-galactoside, quercetin-3-glucoside (1 mg/mL each) (**A**,**B**) and kale water extracts (**C**,**D**), which were detected at 270 and 520 nm.

Moreover, HPLC/DAD/ESI-MS analysis has revealed that standard C3G, K3R, CPG, P3G, M3G and Q3G, as detected at 270 nm, were eluted at the retention time values of 31.618, 33.643, 37.511, 41.165, 43.876 and 47.840 min, respectively (Figure 3A), and all of them except Q3G were also detected at 520 nm (Figure 3B). In contrast, many phenolic compounds in the kale granules, including the six compounds, were eluted at the equivalent time points and detected at 270 nm (Figure 3C). In comparison with the six standards measured at 270 nm, 1 g of the kale granules contained 0.34 mg C3G, 1.15 mg K3R, 0.75 mg CPG, <0.20 mg P3G, 3.15 M3G and 1.85 mg Q3G. Nonetheless, anthocyanins, as measured at 520 nm, were not detected in the kale granules (Figure 3D). Nevertheless, the TAC, as measured with the colorimetric method, was very low (Table 1). Together, phenolics, flavonoids and anthocyanins, which include C3G, K3R, CPG, P3G and M3G, that existed in the kale extract may contribute to its antioxidant, anti-inflammatory and anti-pain properties.

### 3.3. Participant Information

A total of twenty subjects (eight males and twelve females) were enrolled in this study, with an average age of 19.95 ± 1.57 years (a range of 18–23 years) (Table 2). The results were further assessed using the Mann–Whitney test. No significant differences were observed between the two treatment groups for the two molar extractions regarding the duration of the procedures (680 ± 186 vs. 793 ± 358 s).

### 3.4. Pain Intensity

**Figure 4:**
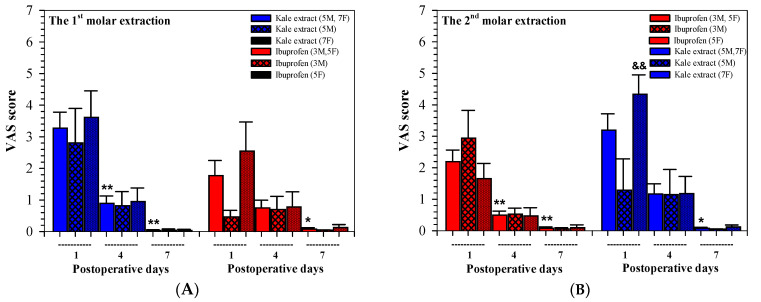
VAS values assessed among twenty participants (8 males, 12 females) who received kale extract (500 mg ACN equivalent) and ibuprofen (400 mg) for 7 days after the 1st (**A**) and 2nd (**B**) molar extractions. VAS scoring from 0 = no pain to 10 = unbearable pain. Accordingly, the data are expressed as median ± SEM values. Paired Student’s t and Wilcoxon test were used to analyze the resulting data, * *p* < 0.05 and ** *p* < 0.01 when compared between time points. Paired Student’s t and Mann–Whitney U tests were used to analyze the resulting data, ^&&^ *p* < 0.01 when compared between the two treatments. Abbreviations: ACN = anthocyanin, SEM = standard error of the mean, VAS = visual analogue score.

As is shown in Figure 4A,B, the VAS values of the male and female groups, as well as those of the 1st and 2nd surgeries, were decreased at 7 days, postoperatively, but no significant differences were observed between the two groups. Interestingly, the VAS values were decreased more by the kale extract than by ibuprofen following the two extractions; however, the degree of efficacy was determined to be predominant after the first extraction.

As is shown in Table 3, almost all VAS values measured on postoperative days 1, 4 and 7 were not significantly different when comparisons were made between male and female participants who were given both kale extract and ibuprofen. Notably, the 1st and 2nd extractions, with the exception of the values measured on day 1 of the 2nd extraction, were different in the females when comparisons were made between the kale extract and ibuprofen treatments (*p* = 0.009 by the Student’s *t*-test).

### 3.5. Salivary Biomarker Concentrations

#### 3.5.1. Salivary AA Activity

**Figure 5:**
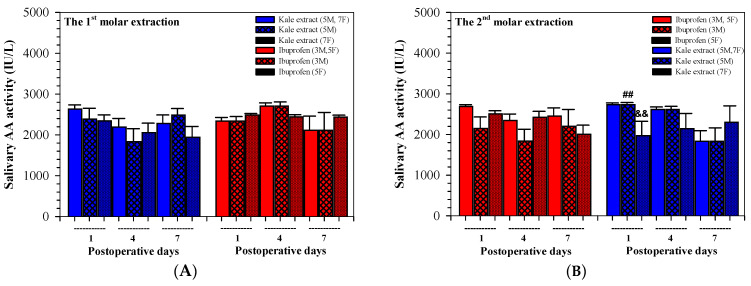
Values of salivary AA activity concentrations analyzed among twenty participants (8 males, 12 females) who received kale extract (500 mg ACN equivalent) and ibuprofen (400 mg) for 7 days after the 1st (**A**) and 2nd (**B**) molar extractions. Accordingly, the data are expressed as median ± SEM values. Paired Student’s t and Mann–Whitney U tests were used to analyze the resulting data, ^##^ *p* < 0.01 when compared between gender, ^&&^ *p* < 0.01 when compared between the two treatments. Abbreviations: AA = α-amylase, ACN = anthocyanin, SEM = standard error of the mean.

Levels of salivary AA activities on day 1 following the first and second tooth extractions were comparable and non-significantly different in the kale extract-treated group and the ibuprofen-treated group. Notably, they were decreased more effectively when treated with kale in accordance with the treatment times in the 2nd tooth extraction (Figure 5A,B and Table 4).

Remarkably, salivary AA activities were compared after the two extractions, amongst genders and/or treatments, and were not determined to be significantly different. Nonetheless, differences were found between males and females in the kale extract group on day 1 after the 2nd extraction (*p* = 0.003 by the student’s *t*-test) and between the two female treatment groups (*p* = 0.001 by the Student’s *t*-test).

#### 3.5.2. Salivary MMP-9

**Figure 6:**
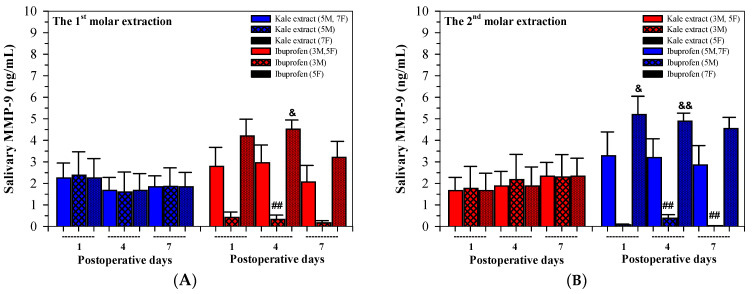
Values of salivary MMP-9 concentrations analyzed among twenty participants (8 males, 12 females) who received kale extract (500 mg ACN equivalent) and ibuprofen (400 mg) for 7 days after the 1st (**A**) and 2nd (**B**) molar extractions. Accordingly, the data are expressed as median ± SEM values. Paired Student’s t and Mann–Whitney U tests were used to analyze the resulting data, ^##^ *p* < 0.01 when compared between gender, ^&^ *p* < 0.05 and ^&&^ *p* < 0.01 when compared between the two treatments. Abbreviations: ACN = anthocyanin, MMP-9 = matrix metalloproteinase 9, SEM = standard error of the mean.

As is shown in Figure 6A,B, following the first tooth extractions, the levels of salivary MMP-9 concentrations were comparable on day 1 in both the kale extract and ibuprofen treatment groups, while the levels were lowered on days 4 and 7 in the kale treatment group and on day 7 in the ibuprofen treatment group. After the second tooth extraction, the levels of salivary MMP-9 concentrations were decreased by the kale treatment but increased by the ibuprofen treatment in accordance with the treatment times.

Interestingly, significant differences in salivary MMP-9 values were observed on days 1, 4 and 7 after the 1st and 2nd molar extractions when comparisons were made between the genders of the patients, of which the values were lower in males than females (Table 5). In addition, differences were only found in female participants who had received either the kale extract or ibuprofen.

#### 3.5.3. Salivary TGF-β_2_

**Figure 7:**
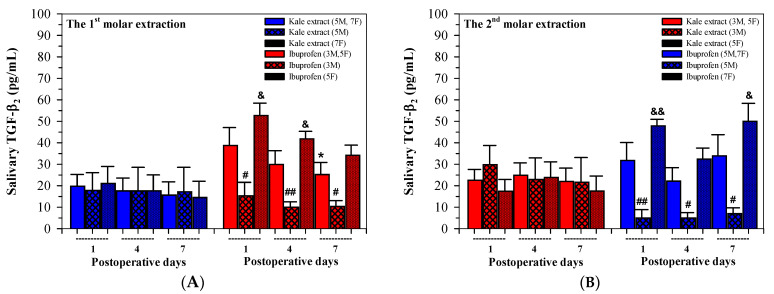
Values of salivary TGF-β_2_ concentrations analyzed among twenty participants (8 males, 12 females) who received kale extract (500 mg ACN equivalent) and ibuprofen (400 mg) for 7 days after the 1st (**A**) and 2nd (**B**) molar extractions. Accordingly, the data are expressed as median ± SEM values. Paired Student’s t and Mann–Whitney U tests were used to analyze the resulting data, which ^#^ *p* < 0.05 and ^##^ *p* < 0.01 when compared between gender, ^&^ *p* < 0.05 and ^&&^ *p* < 0.01 when compared between the two treatments, * *p* < 0.05 when compared between time points. Abbreviations: ACN = anthocyanin, SEM = standard error of the mean, TGF-β2 = transforming growth factor-beta2.

In addition, following the first tooth extractions, levels of salivary TGF-β_2_ concentration were decreased on days 4 and 7 in both the kale extract and ibuprofen treatment groups, whereas salivary TGF-β_2_ levels were not changed on days 4 and 7 in both the kale extract and ibuprofen treatment groups following the second tooth extraction (Figure 7A,B).

Consistently, significant differences of salivary TGF-β_2_ values were also observed on days 1, 4 and 7 after the 1st and 2nd molar extractions when comparisons were made between the genders of the patients, of which the values were lower in males that females, while significant differences were only found in female participants who had received the two treatments (Table 6).

### 3.6. Correlation Among the VAS, Salivary α-Amylase, MMP-9 or TGF-β_2_

**Figure 8:**
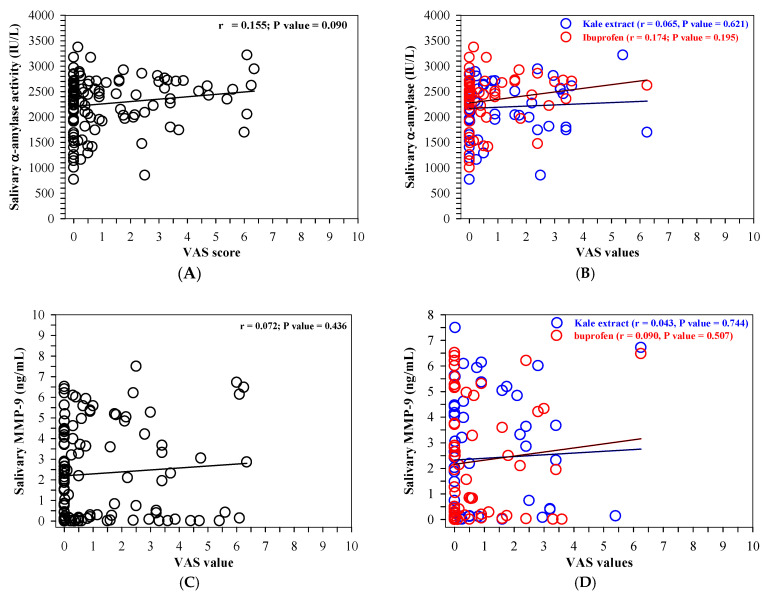

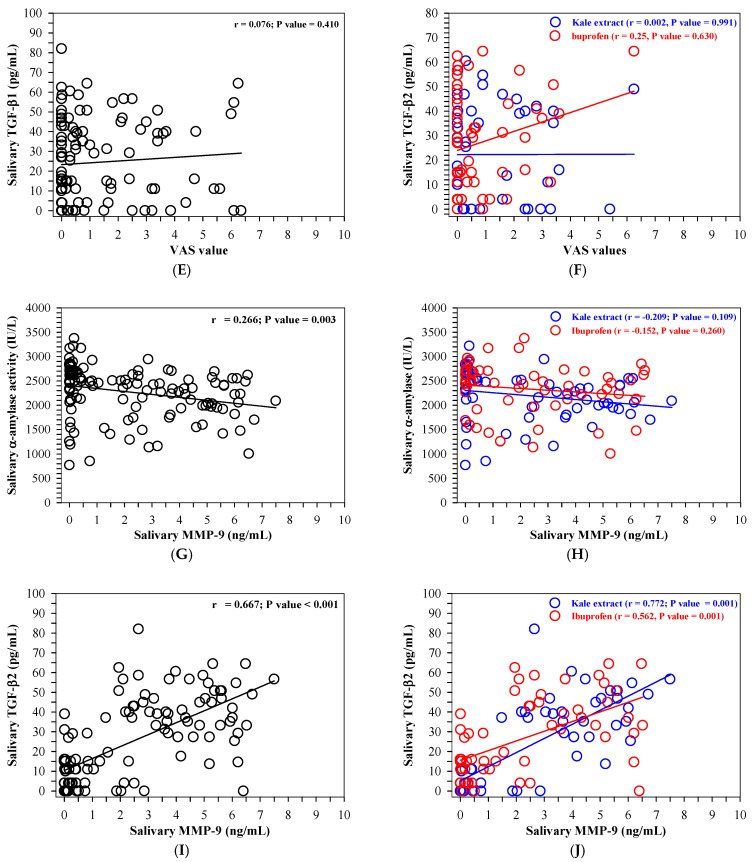

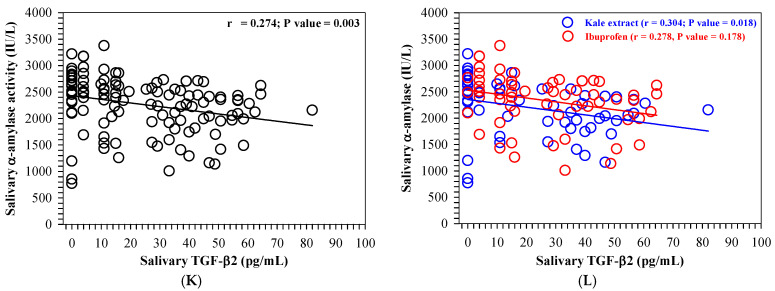
Correlation between the VAS values and α-amylase (**A**,**B**), MMP-9 (**C**,**D**) or TGF-β2 values (**E**,**F**); the MMP-9 and α-amylase values (**G**,**H**) or TGF-β2 (**I**,**J**); TGF-β2 and α-amylase values (**K**,**L**). All the values were measured in participants A–T who had received ibuprofen (400 mg) and the kale extract (500 mg ACN equivalent) (n = 20 each) for 7 days. Accordingly, the data are expressed as median±SEM values. Pearson linear correlation coefficient (r) values for various paired variables were determined. Abbreviations: ACN = anthocyanin, MMP-9 = matrix metalloproteinase 9, SEM = standard error of the mean, TGF-β_2_ = transforming growth factor-beta2.

Of the included patients (n = 20), the Pearson correlation (r) and correlation coefficient (r^2^), along with any statistically significant correlations, were determined among the VAS values and the salivary biomarker concentrations and are shown in Figure 8A–L. The r value indicates the strength of the linear relationship, of which 0.3 < r^2^ < 0.5 demonstrates a moderate relationship, 0.5 < r^2^ < 0.8 a strong relationship and 0.8 < r^2^ < 1.0 a very strong relationship. A *p*-value < 0.05 indicates that a statistically significant relationship is present between that pair of variables. Statistically significant correlations were observed between the AA and the MMP-9 (Figure 8G) the MMP-9 and the TGF-β_2_ (Figure 8I,J) and the AA and the TGF-β_2_ (Figure 8K), whereas no correlations were observed between the other paired biomarkers (Figure 8H,L). There were no correlations between the VAS and any of the biomarkers either (Figure 8A–F).

## 4. Discussion

Postoperation pain, inflammation and discomfort can affect the quality of life of a patient, as well as their daily lives and their degree of satisfaction with treatment. Tissue trauma/injury that occurs during tooth extraction and surgery can cause cellular damage and inflammation in the oral cavity [59]. Phospholipase A2 catalyzes the hydrolysis of cell membrane glycerophospholipids to release arachidonate that will be further catalyzed by cyclooxygenases (COXs) and generate thromboxane and prostaglandins (PGs) [60]. Therefore, NSAIDS are required to effectively manage acute and chronic pain. Among them, ibuprofen (IUPAC name: (RS)-2-(4-(2-methylpropyl)phenyl)propanoic acid) is routinely prescribed to not only suppress the synthesis of powerful inflammatory mediators (e.g., PGs) but also to inhibit platelet attachment and to extend bleeding time [61]. This double-blind, randomized clinical trial was designed to assess and compare the differences between the analgesic and anti-inflammatory effects of oral kale extract capsules (single-dose 500 mg equivalent to ACN) and oral ibuprofen capsules (single-dose 400 mg) after two surgical extractions of the third mandibular molars. Pain catastrophizing scale (PCS) values were determined via a self-assessment type of questionnaire that was employed to examine catastrophizing in normal and clinical populations. This process allows researchers to quantify the patient’s pain experience, using VAS to record an individual’s level of pain intensity.

In the present study, pain perception levels on the operation day (day 1) were not recorded, while VAS scores on day 1 after two molar extractions revealed higher values in the kale extract group when compared with the ibuprofen group. Afterward, the two interventions decreased the level of pain perception to undetectable levels on days 6 and 7.

A diagnosis of periodontal disease is largely achieved through clinical and radiographic investigations that evaluate previously occurring tissue damage and inflammation, but which are not sensitive enough to identify the active phase of the disease and the presence of any symptoms. Therefore, biomarkers in the oral fluid, which can be sensitively and accurately measured, are necessary for diagnosis and follow-up investigations of the disease based on molecular evidence. Preferably, saliva is often used to measure α-amylase, MMP-8, MMP-9 and TGF-β to indicate the state of health of the oral cavity [26,62,63,64,65].

We have previously shown that VAS-assessed pain intensity values and salivary AA levels were significantly higher in patients before mandibular third molar surgery than those after the surgery, while they were significantly and positively correlated with each other [5]. MMPs are a family of zinc-dependent endopeptidase that play a role in physiological and pathological bone remodeling. They are regulated by tissue inhibitors of metalloproteinases (TIMPs) that protect the periodontal ligaments from the MMPs. In periodontal disease, MMPs (e.g., MMP-9) are highly secreted and degrade the extracellular matrix, whereas TIMPs levels can be significantly decreased. For instance, MMP-9 levels in gingival crevicular fluids are increased under conditions of stimulated microgravity (male: 24.12 ± 8.67 ng/mL, females: 26.67 ± 9.41 ng/mL) when compared with normal conditions (male: 12.34 ± 10.23 ng/mL, females: 10.98 ± 11.67 ng/mL) [66]. TGF-β plays an important role in the pathogenesis of oral submucous fibrosis (OSMF), of which salivary TGF-β_1_, β_2_ and β_3_ expressions were increased along with increasing clinical grades of OSMF, thereby advancing the stage of the disease. However, they were decreased after oral submucosal injection of hyaluronidase (1500 IU) [26].

Herein, we have analyzed the water extract obtained from kale (*Brassica oleracea*) leaves and reported on the existence of phenolics, flavonoids and anthocyanins (e.g., cyanidin-3-glucoside, keracyanin-3-rutinoside, callistaphin glucoside, peonidin-3-glucoside, malvidin-3-galactoside and quercetin-3-glucoside) in association with their antioxidative properties. Consistently, Lucic and colleagues have revealed that Croatian kale (*B. oleracea* L. var. acephala DC.) extract was abundant with kaempferol and quercetin and exerted antioxidant and antiproliferative properties on HeLa cells [67]. Interestingly, the ethanolic extract of *B. oleracea* var. capitata contained four desulfoglucosinolates (e.g., desulfo-glucoraphanin, glucoerucin, glucoraphanin and desulfo-glucobrassicin), three flavonols (e.g., kaempherol rhamnoside, isorhamnetin 3-O-acetyl glucoside and quercetin 3-O-6-benzoyl-galactoside), three hydroxycinnamic acid derivatives (e.g., hydroxycinnamic acid p-coumaric acid, caffeoylquinic acid and synapoyl glucoside acid) and two flavones (e.g., apigenin glucoside and apigenin-apiosyl-glucoside). This extract exhibited certain antioxidant, analgesic and anti-inflammatory effects on rats that presented acute and sub-chronic inflammation [68]. Likewise, the methanolic extracts of cauliflower (*B. oleracea* L. var. Botrytis) leaves contain phenolic acids (e.g., ferulic acid, vanillic acid, p-coumaric acid and quercetin) that can relieve gentamicin-induced hepatorenal injury in rats, possibly by the downregulation of interleukin 1beta and through nuclear factor kappaB activity gene expression and protein production [69]. Moreover, cabbage (*B*. *oleracea*) extract exerted a powerful wound healing effect in rats with back injuries by increasing collagen proliferation and consequently stimulating scar tissue maturation [70].

In addition, methanolic and water extracts obtained from curly kale leaves were found to contain phenolics (25.8 and 10.8 mM GAE, respectively), flavonoids (17.9 and 5.3 mM rutin equivalent, RE, respectively) and flavonols (5.8 and 2.1 mM RE, respectively), which potentially presented antioxidant properties (66.5 and 45.4 mM TE, respectively) and inhibited vascular cell adhesion that was induced by tumor necrotic factor alpha (TNF-α) under inflammatory conditions [71]. Moreover, the ethyl acetate extract of Chinese cabbage, which is a closely related *Brassica* spp. to curly kale, exhibited an anti-inflammatory effect on lipopolysaccharide-induced RAW 264.7 cells by inhibiting the production of pro-inflammatory cytokines, including TNF-α and interleukins (ILs) 1 and 6, COXs, nitric oxide synthase and IL-6 gene [72]. Furthermore, glucosinolates are important phytochemicals existing in broccoli, kale and cabbages that exert antioxidant activities by regulating NADPH quinone oxidoreductase 1, glutamate-cysteine ligase and heme oxygenase-1. They also exert anti-inflammatory effects by modulating nuclear factor erythroid 2-related factor 2, pro-inflammatory cytokines, signal transducers and activators of transcription 3 [73]. Recently, we have reported that hot water and 70% ethanolic extracts of curly kale leaves contained phenolics (6.12 and 4.22 mg GAE/g, respectively), flavonoids (0.92 and 0.92 mg QE/g, respectively) and anthocyanins (48.4 and 37.4 μg CE/g, respectively), which exerted antioxidant properties (2.82 and 2.21 mg TE/g, respectively) but were not toxic to human peripheral blood mononuclear cells and fibroblast (HaCaT) cells [74].

In terms of quality control and the nutraceutical properties of kale products, comprehensive microscopic, high-performance thin-layer chromatographic, high-performance liquid chromatographic/mass spectrometric, high-performance liquid chromatography-quadrupole time-of-flight mass spectrometric and spectroscopic methods were used to reveal distinct localization and chemical profiles with relevant quantities of flavonoids (e.g., sinapine isomers, feruloyl and sinapoyl choline derivatives), total lipids, alkaloids and glucosinolates [75]. Since anthocyanins (ACNs) have been reported to be rich in many pigmented plants-derived products, the ACN equivalent amounts of the products have been studied to assess their quality, functionality, efficacy and consistency [76]. Hence, quality assessment of the kale extract product used in this study was based on the total anthocyanin content.

In this randomized, double-blind split-mouth clinical trial, we have investigated patients that had begun consuming a plant-derived product (500 mg ACN equivalent kale extract) and an NSAID (single-dose 400 mg ibuprofen), and vice versa, three times daily for 7 days after two lower molar extractions. Accordingly, significant decreases in the levels of pain perception were assessed through VAS scoring, while an analgesic effect was indicated by salivary α-amylase activity. Furthermore, inflammation was monitored by salivary MMP-9 and TGF-β_2_ values in the kale extract group when compared with members of the control group who had been given ibuprofen. Importantly, a positive correlation was observed between the MMP-9 and TGF-β_2_ values in the saliva collected from patients who had undergone two mandibular molar extractions and had received treatment. Accordingly, this study highlights the applications of relevant physical examinations (VAS values) and biological assays (e.g., AA, MMP-9 and TGF-β_2_) by monitoring the recovery of gingival trauma and lesions after lower mandibular molar extractions.

In terms of the potential limitations, this study involved a small sample size (n = 20) that could have introduced limitations of generalizability and heterogeneity, the risk of random variability, and lowered precision and reliability of the data. In addition, variability in patient responses to the treatments could have been encountered. Likewise, the subjects’ stress and anxiety levels, which may have been caused by a fear of the extraction procedure, could significantly influence their perceived levels of pain. Moreover, only three surrogate biomarkers, such as salivary AA, MMP-9 and TGF-β_2_, were measured. These factors may have affected the reliability of the results. Furthermore, the relatively short study time of this study and the small budget allocated for our clinical trial presented obstacles for post-graduate resident dentists in conducting an effective clinical trial. Thus, this study assessed the patients’ perceived levels of pain using direct subjective VAS, which could potentially have led to exaggerations and inaccuracies in some anxious patients. Moreover, the degree of inflammation of the patients was established using indirect determinations of salivary α-amylase, MMP-9 and TGF-β_2_, which may have varied according to the individual, their specimen stability and between assay values. In terms of recommendations, we suggest that more randomized clinical trials involving larger numbers of participants should be carried out to further validate these findings. In addition, a section of the reliable questionnaire could be dedicated to the patients’ stress and anxiety levels. Likewise, patients could be investigated for extended periods of time, with kale extract products being prescribed for days after the molar extraction procedure. Moreover, the efficacy of both the kale extract and NSAIDs, along with their degree of superiority/inferiority with regard to their analgesic properties, should be investigated clinically in other types of oral surgeries (e.g., dental implants, pathologies and soft tissue surgeries). Furthermore, the outcomes of a combined treatment of kale extract with ibuprofen could be compared with single treatments of the kale extract, ibuprofen or other analgesics.

## 5. Conclusions

The consumption of kale extract, which is rich in phenolics, flavonoids and anthocyanins, in a daily dose of 500 mg of anthocyanin for 7 days, has revealed significant analgesic and anti-inflammatory effects on patients who had undergone impacted mandibular molar surgery. Accordingly, the kale extract was determined to be more potent than ibuprofen. In addition, salivary MMP-9 levels, but not the other parameters, exhibited a positive correlation with salivary TGF-β_2_ values. Therefore, kale extract consumption would reduce the requirement for NSAIDs, suggesting a possible alternative to ibuprofen and providing greater efficacy in the treatment for postoperative patients. As a recommendation, the kale extract treatment is an option worthy of clinical attention in dental surgery. A further investigation involving a larger number of patients is necessary to evaluate the analgesic and anti-inflammatory impacts of the kale extract in surgical procedures, aside from mandibular third molar extraction and surgery.

## Figures and Tables

**Table 1:** Total phenolic, flavonoid and anthocyanin contents and antioxidant activity detected in kale extract granule. Data are expressed as mean ± SD values.

Sample	TPC (mg GAE/g)	TFC (mg QE/g)	TAC (µg CE/g)	Antioxidant Activity (mg TE/g)
Kale extract granule	24.92 ± 0.46	3.09 ± 1.51	204.72 ± 1.71	3.18 ± 0.82

Abbreviations: GAE = gallic acid equivalent, CE = cyanidin-3-glucoside equivalent, QE = quercetin equivalent, TAC = total anthocyanin content, TE = Trolox equivalent, TFC = total flavonoid content, TPC = total phenolic content.

**Table 2:** Information of participants (n = 20) enrolled in the study who had undergone tooth extraction and who were treated with kale extract (500 mg ACN equivalent) or ibuprofen (400 mg). Data are expressed as absolute or mean ± SD values.

Subject	Gender	Age (y)	First Molar Extraction		Second Molar Extraction	
			Duration (sec)	Treatment	Duration (sec)	Treatment
A	F	19	721	ibuprofen	718	kale extract
B	F	19	438	ibuprofen	451	kale extract
C	F	18	723	kale extract	718	ibuprofen
D	M	21	675	kale extract	1941	ibuprofen
E	F	18	411	ibuprofen	824	kale extract
F	F	23	420	kale extract	605	ibuprofen
G	F	20	507	ibuprofen	573	kale extract
H	M	19	751	kale extract	582	ibuprofen
I	F	21	732	ibuprofen	1131	kale extract
J	F	20	657	kale extract	588	ibuprofen
K	M	22	926	ibuprofen	801	kale extract
L	F	19	695	kale extract	425	ibuprofen
M	M	18	1139	kale extract	1070	ibuprofen
N	M	21	907	ibuprofen	1366	kale extract
O	M	23	743	kale extract	682	ibuprofen
P	F	19	635	kale extract	798	ibuprofen
Q	F	20	624	kale extract	652	ibuprofen
R	M	18	522	ibuprofen	484	kale extract
S	M	21	520	kale extract	611	ibuprofen
T	F	20	857	kale extract	833	ibuprofen
	8 M, 12 F	19.9 ± 1.6	680 ± 186	8 ibuprofen 12 kale extract	793 ± 358	12 ibuprofen 8 kale extract

Abbreviations: ACN = anthocyanin, F = female, M = male.

**Table 3:** Significant differences of VAS values (mean ± SD) assessed on days 1, 4 and 7 among post-molar extraction participants A–T (8 males and 12 females) receiving kale extract (500 mg ACN equivalent) and ibuprofen (400 mg) for 7 days. Paired Student’s t and Wilcoxon test were used to analyze the resulting data.

1st Lower Molar Extraction				2nd Lower Molar Extraction			
Intervention	Day	VAS Value	*p*-Value	Intervention	Day	VAS Value	*p*-Value
Kale extract (n = 12)	1	3.28 ± 0.50	-	Ibuprofen (n = 12)	1	2.19 ± 1.37	-
	1/4	0.90 ± 0.23	0.005		1/4	0.50 ± 0.13	0.008
	1/7	0.04 ± 0.02	0.002		1/7	0.08 ± 0.04	0.008
Kale extract (5 M, 7 F)	1	2.81 ± 1.09, 3.61 ± 0.84	0.565	Ibuprofen (3 M, 5 F)	1	2.94 ± 0.88, 1.66 ± 0.48	0.198
	4	0.82 ± 0.45, 0.95 ± 0.43	1.000		4	0.53 ± 0.19, 0.47 ± 0.26	0.870
	7	0.04 ± 0.04, 0.04 ± 0.04	0.901		7	0.06 ± 0.04, 0.09 ± 0.09	0.454
Ibuprofen (n = 8)	1	1.77 ± 0.48	-	Kale extract (n = 12)	1	3.20 ± 0.52	-
	1/4	0.75 ± 0.24	0.091		1/4	1.17 ± 0.32	0.052
	1/7	0.08 ± 0.04	0.028		1/7	0.08 ± 0.03	0.027
Ibuprofen (3 M, 5 F)	1	0.47 ± 0.26, 2.55 ± 1.09	0.205	Kale extract (5 M, 7 F)	1	1.29 ± 1.29, 4.34 ± 0.73	0.064
	4	0.70 ± 0.53 *, 0.78 ± 0.56	0.579		4	1.15 ± 1.03, 1.18 ± 0.64	0.980
	7	0.00 ± 0.00, 0.13 ± 0.11	0.242		7	0.00 ± 0.00, 0.12 ± 0.07	0.237
8 M (kale extract, ibuprofen)	1	2.81 ± 1.09, 0.47 ± 0.26	0.053	12 F (ibuprofen, kale extract)	1	1.66 ± 0.48, 4.34 ± 0.73	0.009
	4	0.82 ± 0.45, 0.70 ± 0.53	0.872		4	0.47 ± 0.26, 1.18 ± 0.64	0.435
	7	0.04 ± 0.04, 0.00 ± 0.00	0.439		7	0.09 ± 0.09, 0.12 ± 0.07	0.454
12 F (kale extract, ibuprofen)	1	3.61 ± 0.84, 2.55 ± 1.09	0.450	8 M (ibuprofen, kale extract)	1	2.94 ± 0.88, 1.29 ± 1.29	0.314
	4	0.95 ± 0.43, 0.78 ± 0.56	0.740		4	0.53 ± 0.19, 1.15 ± 1.03	0.764
	7	0.04 ± 0.04, 0.13 ± 0.11	0.337		7	0.06 ± 0.04, 0.00 ± 0.00	0.237

Abbreviations: ACN = anthocyanin, VAS = visual analogue scale, * *p* < 0.05 when compared between time points.

**Table 4:** Significant differences of salivary AA activity values (mean ± SEM) assessed on days 1, 4 and 7 among post-molar extraction participants A–T (8 males and 12 females) receiving kale extract (500 mg ACN equivalent) and ibuprofen (400 mg) for 7 days. Paired Student’s t and Mann–Whitney U tests were used to analyze the resulting data.

1st Lower Molar Extraction				2nd Lower Molar Extraction			
Intervention	Day	AA Activity (IU/L)	*p*-Value	Intervention	Day	AA Activity (IU/L)	*p*-Value
Kale extract (n = 12)	1	2632 ± 101	-	Ibuprofen (n = 8)	1	2686 ± 46	-
	1/4	2193 ± 210	0.097		1/4	2347 ± 154	0.665
	1/7	2282 ± 207	0.376		1/7	2452 ± 199	0.642
Kale extract (5M, 7F)	1	2389 ± 262, 2346 ± 41	0.879	Ibuprofen (3M, 5F)	1	2149 ± 280, 2508 ± 78	0.167
	4	1831 ± 319, 2054 ± 234	0.578		4	1840 ± 286, 2425 ± 142	0.052
	7	2485 ± 159, 1942 ± 264	0.145		7	2201 ± 412, 2008 ± 222	0.873
Ibuprofen (n = 8)	1	2341 ± 86	-	Kale extract (n = 12)	1	2732 ± 42	-
	1/4	2708 ± 79	0.225		1/4	2615 ± 60	0.138
	1/7	2114 ± 436	0.472		1/7	1830 ± 263	0.158
Ibuprofen (3M, 5F)	1	2341 ± 140, 2488 ± 47	0.270	Kale extract (5M, 7F)	1	2732 ± 62, 1972 ± 109	0.003
	4	2708 ± 222, 2444 ± 80	0.112		4	2615 ± 98, 2144 ± 142	0.059
	7	2114 ± 562, 2436 ± 82	0.478		7	1830 ± 429, 2302 ± 81	0.204
8M (kale extract, ibuprofen)	1	2389 ± 262, 2341 ± 140	0.899	12F (ibuprofen, kale extract)	1	2654 ± 77, 1972 ± 109	0.001
	4	1831 ± 319, 2708 ± 222	0.091		4	2487 ± 143, 2144 ± 142	0.129
	7	2485 ± 159, 2114 ± 563	0.453		7	2131 ± 222, 2302 ± 81	0.547
12F (kale extract, ibuprofen)	1	2347 ± 141, 2488 ± 48	0.435	8M (ibuprofen, kale extract)	1	2148 ± 280, 2732 ± 62	0.180
	4	2054 ± 235, 2444 ± 80	0.208		4	1840 ± 286, 2615 ± 98	0.094
	7	1942 ± 265, 2436 ± 82	0.372		7	2201 ± 412, 1830 ± 429	0.578

Abbreviation: ACN = anthocyanin, AA = α-amylase, IU = international unit.

**Table 5:** Significant differences of salivary MMP-9 values (mean ± SEM) assessed on days 1, 4 and 7 among post-molar extraction participants A–T (8 males and 12 females) receiving kale extract (500 mg ACN equivalent) and ibuprofen (400 mg) for 7 days. Paired Student’s t and Mann–Whitney U tests were used to analyze the resulting data.

1st Lower Molar Extraction				2nd Molar Extraction			
Intervention	Day	MMP-9 (ng/mL)	*p*-Value	Intervention	Day	MMP-9 (ng/mL)	*p*-Value
Kale extract (n = 12)	1	2.25 ± 0.69	-	Ibuprofen (n = 8)	1	1.67 ± 0.61	-
	1/4	1.67 ± 0.60	0.286		1/4	1.88 ± 0.68	0.913
	1/7	1.84 ± 0.51	0.126		1/7	1.33 ± 0.64	0.401
Kale extract (5 M, 7 F)	1	1.87 ± 1.16, 2.52 ± 0.91	0.462	Ibuprofen (3 M, 5 F)	1	1.78 ± 1.22, 1.59 ± 0.68	0.368
	4	1.54 ± 0.99, 1.76 ± 0.82	1.000		4	2.40 ± 1.39,1.50 ± 0.69	0.808
	7	1.67 ± 1.00, 1.96 ± 0.58	0.935		7	1.26 ± 0.54, 3.10 ± 0.96	0.167
Ibuprofen (n = 8)	1	2.79 ± 0.89	-	Kale extract (n = 12)	1	3.28 ± 1.11	-
	1/4	2.95 ± 0.83	0.812		1/4	3.20 ± 0.87	0.844
	1/7	1.84 ± 0.51	0.467		1/7	2.86 ± 0.90	0.117
Ibuprofen (5 M, 5 F)	1	0.42 ± 0.32, 4.21 ± 0.92	0.023	Kale extract (5 M, 7 F)	1	0.08 ± 0.04, 5.20 ± 1.01	0.009
	4	0.33 ± 0.26, 4.53 ± 0.50	0.001		4	0.38 ± 0.21, 4.89 ± 0.44	0.001
	7	0.16 ± 0.13, 3.20 ± 0.89	0.042		7	0.03 ± 0.00, 4.55 ± 0.61	0.001
8 M (kale extract, ibuprofen)	1	1.87 ± 1.16, 0.42 ± 0.32	0.264	12 F (ibuprofen, kale extract)	1	1.59 ± 0.68, 5.20 ± 1.01	0.011
	4	1.54 ± 0.99, 0.33 ± 0.26	0.127		4	1.50 ± 0.69, 4.89 ± 0.44	0.004
	7	1.67 ± 1.00, 0.16 ± 0.13	0.188		7	3.10 ± 0.96, 4.55 ± 0.61,	0.274
12 F (kale extract, ibuprofen)	1	2.52 ± 0.91, 4.21 ± 0.92	0.234	8 M (ibuprofen, kale extract)	1	1.78 ± 1.22, 0.08 ± 0.04	1.000
	4	1.76 ± 0.82, 4.53 ± 0.50	0.026		4	2.40 ± 1.39, 0.38 ± 0.21	0.655
	7	1.96 ± 0.58, 3.20 ± 0.89	0.247		7	1.26 ± 0.54, 0.03 ± 0.00	0.177

Abbreviations: ACN = anthocyanin, MMP-9 = matrix metalloproteinase 9.

**Table 6:** Significant differences of salivary TGF-β_2_ values (mean ± SEM) assessed on days 1, 4 and 7 among post-molar extraction participants A–T (8 males and 12 females) receiving kale extract (500 mg ACN equivalent) and ibuprofen (400 mg) for 7 days. Paired Student’s t and Mann–Whitney U tests were used to analyze the resulting data.

1st Lower Molar Extraction				2nd Molar Extraction			
Intervention	Day	TGF-β2 (pg/mL)	*p*-Value	Intervention	Day	TGF-β2 (pg/mL)	*p*-Value
Kale extract (n = 12)	1	19.75 ± 5.50	-	Ibuprofen (n = 8)	1	22.58 ± 5.01	-
	1/4	17.60 ± 5.98	0.310		1/4	24.91 ± 5.68	0.067
	1/7	15.62 ± 6.17	0.123		1/7	22.03 ± 6.17	0.706
Kale extract (5 M, 7 F)	1	17.84 ± 8.27, 21.11 ± 7.86	0.785	Ibuprofen (3 M, 5 F)	1	29.76 ± 9.00, 17.45 ± 5.45	0.243
	4	17.58 ± 10.98, 17.61 ± 7.43	0.862		4	26.36 ± 10.06, 23.88 ± 7.25	0.841
	7	17.19 ± 11.38, 14.51 ± 7.57	0.928		7	28.26 ± 11.53, 17.57 ± 6.96	0.512
Ibuprofen (n = 8)	1	38.71 ± 8.40	-	Kale (n = 12)	1	31.80 ± 8.31	-
	1/4	29.87 ± 6.42	0.106		1/4	22.14 ± 6.27	0.688
	1/7	25.22 ± 5.61	0.043		1/7	33.87 ± 9.92	0.894
Ibuprofen (3 M, 5 F)	1	15.33 ± 8.09, 52.73 ± 6.85	0.014	Kale extract (5 M, 7 F)	1	5.00 ± 5.00, 47.88 ± 3.63	0.001
	4	10.00 ± 3.21, 41.79 ± 4.17	0.002		4	5.00 ± 3.21, 32.42 ± 6.04	0.017
	7	10.33 ± 3.48. 34.15 ± 5.59	0.023		7	7.00 ± 3.51, 50.00 ± 9.93	0.019
8 M (kale extract, ibuprofen)	1	17.84 ± 8.27, 15.33 ± 8.09	0.848	12 F (ibuprofen, kale extract)	1	17.45 ± 5.45, 47.88 ± 3.63	0.002
	4	17.58 ± 10.98, 10.00 ± 3.21	0.628		4	23.88 ± 7.25, 32.42 ± 6.04	0.415
	7	17.19 ± 11.38, 10.33 ± 3.48	0.672		7	17.57 ± 6.96, 50.00 ± 9.93	0.020
12 F (kale extract, ibuprofen)	1	21.11 ± 7.86, 52.73 ± 6.85	0.017	8 M (ibuprofen, kale extract)	1	29.76 ± 9.00, 5.00 ± 5.00	0.960
	4	17.61 ± 7.43, 41.79 ± 4.17	0.030		4	26.36 ± 10.06, 5.00 ± 3.21	0.168
	7	14.51 ± 7.57, 34.15 ± 5.59	0.083		7	28.26 ± 11.53, 7.00 ± 3.51	0.134

Abbreviations: ACN = anthocyanin, TGF-β2 = transforming growth factor beta2.

## Data Availability

The original contributions presented in the study are included in the article, further inquiries can be directed to the corresponding authors.
